# Fatty Acid Composition and Oxidative Changes in Pecan and Walnut Oils from Eastern Europe

**DOI:** 10.3390/molecules31173093

**Published:** 2026-09-03

**Authors:** Daniela Sabina Poşta, Ileana Cocan, Ersilia Alexa, Adina Berbecea, Olimpia Alina Iordănescu, Ion Roşca, Orsolya Borsai, Sándor Rózsa, Isidora Radulov

**Affiliations:** 1 Faculty of Engineering and Applied Technologies, University of Life Sciences King Mihai I from Timisoara, 119 Calea Aradului, 300645 Timisoara, Romania; danielaposta@usvt.ro (D.S.P.); olimpiaiordanescu@usvt.ro (O.A.I.); 2 Faculty of Food Engineering, University of Life Sciences King Mihai I from Timisoara, 119 Calea Aradului, 300645 Timisoara, Romania; ersiliaalexa@usvt.ro; 3 Faculty of Agriculture, University of Life Sciences King Mihai I from Timisoara, 119 Calea Aradului, 300645 Timisoara, Romania; adina_berbecea@usvt.ro; 4 Botanical Garden of the National Institute Alexandru Ciubotaru, MD–2002 Chisinau, Moldova; ion.rosca@sti.usm.md; 5 Faculty of Horticulture and Business in Rural Development, University of Agricultural Sciences and Veterinary Medicine Cluj–Napoca, 3–5 Manastur St., 400374 Cluj–Napoca, Romania; orsolya.borsai@usamvcluj.ro

**Keywords:** *Carya illinoinensis*, *Juglans regia*, fatty acid composition, GC–MS, TBARS, peroxide value, lipid oxidation, proximate composition, Eastern Europe, hot pressing

## Abstract

*Carya illinoinensis* (pecan) has recently emerged as a promising oilseed crop in Eastern Europe; however, its oil has not been systematically characterized under regional growing conditions or directly compared with that of the established nut crop, *Juglans regia* (walnut). This study evaluated the proximate composition, fatty acid profile, and short-term oxidative stability of oils from six pooled sample groups, comprising three pecan and three walnut composite samples collected from Romania and the Republic of Moldova. Oils were extracted by hot pressing, kernel composition was determined using standard official (AOAC) methods, fatty acid composition was analyzed by gas chromatography–mass spectrometry (GC–MS), and oxidative stability was assessed by peroxide value (PV) and thiobarbituric acid reactive substances (TBARS) during 20 days of storage. Romanian pecan oils contained 67–68% total fat and were rich in linoleic acid (55–57%), whereas walnut oils contained 59–61% fat and were dominated by oleic acid (57–58%). The PNC composite sample exhibited an oleic acid-dominant profile that differed from those of the other two pecan samples. Because cultivar identity, genotype, and environmental variables were not determined, the cause of this difference cannot be established from the present study. All samples remained below the Codex Alimentarius PV limit (3.0 meq O_2_/kg). TBARS changes differed among the analyzed composite oils; however, these differences cannot be attributed solely to fatty acid composition because antioxidant constituents were not measured. Oleic and linoleic acids were strongly and inversely correlated (Pearson r = −0.9998, *p* < 0.001). These findings provide the first comprehensive characterization of pecan oil produced under Eastern European conditions and demonstrate compositional variation among the analyzed composite samples, although the factors responsible for this variation could not be determined.

## 1. Introduction

Pecan (*Carya illinoinensi* K. Koch) is a deciduous tree species native to the Mississippi River basin and northern Mexico, where it has long been cultivated for its high-value edible kernels and oil. Although initially introduced into Eastern Europe as an ornamental species, pecan has recently attracted increasing interest as a potential oilseed crop. Romania and the Republic of Moldova now host their first mature commercial-scale plantations, reflecting the broader expansion of pecan cultivation driven by increasing demand for tree nuts and high-quality vegetable oils [1]. Despite this growing interest, the chemical composition and oxidative characteristics of pecan oil produced under Eastern European climatic conditions remain unknown. Consequently, no analytical data are currently available to evaluate whether regional environmental conditions influence the lipid composition of pecan oil. Walnut (*Juglans regia* L.) represents the most appropriate comparator because it is widely cultivated throughout the region and its oil has been extensively characterized with respect to fatty acid composition and physicochemical properties [2,3,4]. However, to our knowledge, no previous study has directly compared the lipid composition and oxidative stability of pecan and walnut oils produced under comparable Eastern European conditions using identical analytical procedures.

Pecan kernels are characterized by an exceptionally high lipid content, typically ranging from 65% to 75% of dry weight [5,6]. Studies made by Toro-Vazques et al. [7], Rivera-Rangel et al. [8], and Ferrari et al. [9] in North and South America consistently report that pecan oil is rich in unsaturated fatty acids, with oleic acid (C18:1) generally representing the predominant component. The relative proportions of monounsaturated fatty acids (MUFAs) and polyunsaturated fatty acids (PUFAs) largely determine both the nutritional quality and oxidative stability of edible oils. Oils with a higher MUFA content generally exhibit greater oxidative stability than PUFA-rich oils, resulting in enhanced shelf life and improved stability during storage and processing, as reported by Vaidya and Eun [10]. Conversely, linoleic acid (C18:2), the principal omega-6 PUFA in pecan oil, contributes substantially to its nutritional value [11,12,13,14]. In addition to their fatty acid composition, tree nut oils contain bioactive compounds, including tocopherols, phytosterols, and phenolic compounds, which further contribute to their antioxidant properties [5]. Consequently, comprehensive characterization of fatty acid composition together with oxidative stability is essential for evaluating the quality of pecan oil.

Tree nuts are widely recognized as components of healthy dietary patterns because their favorable lipid composition and bioactive phytochemicals have been associated with beneficial cardiovascular and metabolic effects [5,15,16,17,18,19]. These health-promoting properties arise from the combined contribution of unsaturated fatty acids and naturally occurring antioxidant compounds. Therefore, detailed characterization of the lipid profile and oxidative behavior of tree nut oils remains important not only from a nutritional perspective but also for assessing their technological quality and commercial value.

Although the general composition of pecan oil has been extensively investigated, its fatty acid profile varies considerably among cultivars and production environments. Toro–Vazquez et al. [7] reported oleic acid concentrations ranging from approximately 55% to 75% in native Mexican pecan populations, whereas Rivera–Rangel et al. [8] demonstrated that cultivar-dependent variation in the C18:1/C18:2 ratio resulted in measurable differences in oxidative stability. Similar observations were reported in Uruguayan orchards by Ferrari et al. [9], confirming that environmental conditions substantially influence lipid composition. Collectively, these studies indicate that both genotype and growing environment contribute to the fatty acid profile of pecan oil, emphasizing the importance of regional compositional studies.

Fatty acid desaturase 2 (FAD2), a Δ12-fatty acid desaturase, catalyzes the conversion of oleic acid into linoleic acid, and previous studies indicate that its activity may be influenced by genotype, developmental stage, and temperature [20,21,22]. This mechanism provides relevant biological context for variation in C18:1 and C18:2 proportions. However, FAD2 expression, genotype, cultivar identity, phenological observations, and meteorological data corresponding to kernel development and harvest were not available in the present study. Consequently, the present study does not evaluate climatic regulation of FAD2 activity; the FAD2 pathway is mentioned only as biological context derived from previous literature and not as a mechanism demonstrated by the present results [23,24].

Despite the increasing cultivation of pecan in Eastern Europe, no analytical characterization of its oil composition has yet been reported. In contrast, walnut oil produced in Romania has been extensively characterized with respect to its fatty acid composition, physicochemical properties, and oxidative stability in previous studies by Iordănescu et al. [2], Trandafir et al. [25], and Trandafir et al. [26], while the proximate composition of Romanian walnut kernels has also been documented. This lack of comparable analytical information represents an important knowledge gap in the characterization of pecan oil produced under Eastern European conditions.

To address this gap, the present study directly compared the lipid composition and oxidative stability of pecan and walnut oils produced under comparable environmental conditions in Romania and the Republic of Moldova. Six pooled sample groups, comprising three pecan and three walnut populations, were collected from identical geographical locations and analyzed using the same extraction procedure and analytical methodology. Fatty acid composition was determined by gas chromatography–mass spectrometry (GC–MS), while oxidative stability was evaluated using peroxide value (PV) and thiobarbituric acid reactive substances (TBARS) as complementary indicators of primary and secondary lipid oxidation. Specifically, the objectives of this study were to (i) compare the fatty acid composition of pecan and walnut oils from three Eastern European production sites, (ii) evaluate their short-term oxidative stability, and (iii) explore compositional and oxidative differences among the six analyzed composite samples.

## 2. Results

### 2.1. Proximate Composition

Table 1 shows proximate composition across the six groups. Analysis of variance (ANOVA) indicated significant between-group differences for all parameters (moisture: F5,12 = 18.4, *p* < 0.001; crude protein: F5,12 = 87.3, *p* < 0.001; total fat: F5,12 = 143.6, *p* < 0.001).

Moisture ranged from 2.17% (WCN) to 3.99% (PNC). PNC showed the highest moisture content and the lowest fat content in this dataset, but the present design does not allow attribution of these differences to specific climatic factors. These values bracket the 2.1–6.4% range reported by Venkatachalam et al. [27] for 16 North American pecan cultivars and fall within the 2.1–3.99% observed by Siebeneichler et al. [28] for 26 Brazilian cultivars. Flores-Córdova et al. [29] reported moisture contents of 2.94% and 3.70% in the edible portions of the Wichita and Western Schley pecan cultivars, respectively, values comparable to those observed in the present study.

Among the analyzed composite samples, protein values were lower in the three pecan samples (8.4–10.3%) than in the three walnut samples (12.2–14.4%). Wakeling et al. [6] found 8.5–9.8% for Australian pecan cultivars; Yerlikaya et al. [30] found 10.6–18.2% for Turkish *Juglans regia* genotypes. Kafkas et al. [31] confirmed a comparable protein range (7.6–13.4%) across diverse walnut genotypes from the USA. Romanian walnut genotypes have also shown wide variation in protein content [2], while other Romanian walnut material exhibited substantial variability in phenolic content and antioxidant activity [32].

Total fat showed the largest difference among the six analyzed composite samples (F5,12 = 143.6). Based on the analytical measurements, PNL (68.2%) and PNCN (67.4%) had higher total fat values than the three analyzed walnut composite samples (59.3–61.3%, *p* < 0.05), in line with the 65–75% range documented for American and Australian pecan cultivars [6,27]. Scapinello et al. [31] reported fat content of 64–71% for cold-pressed pecan oils. PNC (51.9%) was the statistical outlier, significantly below PNL and PNCN (*p* < 0.001) and even below the walnut samples. Ferrari et al. [9] attributed comparable within-species fat variation in Uruguayan pecan orchards to soil type and year-to-year rainfall. Poletto et al. [32] similarly documented fat content ranging from 52% to 74% across 26 Brazilian pecan accessions, attributing the spread to pedoclimatic variation across sites. Caloric value tracked fat content almost perfectly (r = +0.998, *p* < 0.001; Table 1).

### 2.2. Fatty Acid Composition

Table 2 presents the GC–MS fatty acid data. ANOVA detected significant between-group differences for all 12 identified fatty acids except margaric acid (C17:0, *p* = 0.21).

Among the analyzed composite samples, WCN and WC contained 57.9% and 57.1% C18:1n–9 (Δ9), respectively, and 30.8% and 32.3% C18:2n–6 (Δ9,12). PNL and PNCN contained higher relative proportions of C18:2n–6, at 55.5% and 56.8%, and lower relative proportions of C18:1n–9, at 31.5% and 30.9%, respectively. Similar sample-level variation in the relative proportions of oleic and linoleic acids has been reported in previous studies of pecan and walnut oils [6,8,9]. WL contained 22.5% C18:1n–9 and 64.4% C18:2n–6, whereas PNC contained 56.8% C18:1n–9 and 32.0% C18:2n–6. The relative C18:1 and C18:2 proportions of PNC were similar to those observed in WCN and WC. However, cultivar identity, genotype, soil characteristics, and meteorological parameters were not available; therefore, the factors responsible for these sample-level differences cannot be determined. α-linolenic acid (C18:3n–3) was detected only in WL (0.16%) and PNL (0.02%). PNC had the highest moisture value and the lowest total fat value among the three pecan composite samples. The present experimental design does not allow these observations to be attributed to rainfall, soil conditions, genotype, cultivar, or other specific factors; PNC converged with the walnut fatty acid profile. Alpha-linolenic acid (C18:3n–3) appeared only in WL (0.16%) and PNL (0.02%), which may be related to processing conditions, although this study did not include a temperature-controlled comparison [33].

Across the six composite-sample means, the relative proportions of C18:1 and C18:2 were strongly inversely associated (Pearson r = −0.9998, *p* < 0.001; Spearman ρ = −0.943, *p* = 0.005). However, both variables were expressed as percentages of the same total and together accounted for approximately 80–90% of the relative fatty acid profile. Therefore, the strength of this inverse association is influenced by the closed compositional structure of the data and should be interpreted as a descriptive relationship among relative fatty acid proportions rather than as evidence of a direct biological or biosynthetic mechanism.

### 2.3. Oxidative Stability

**Figure 1:**
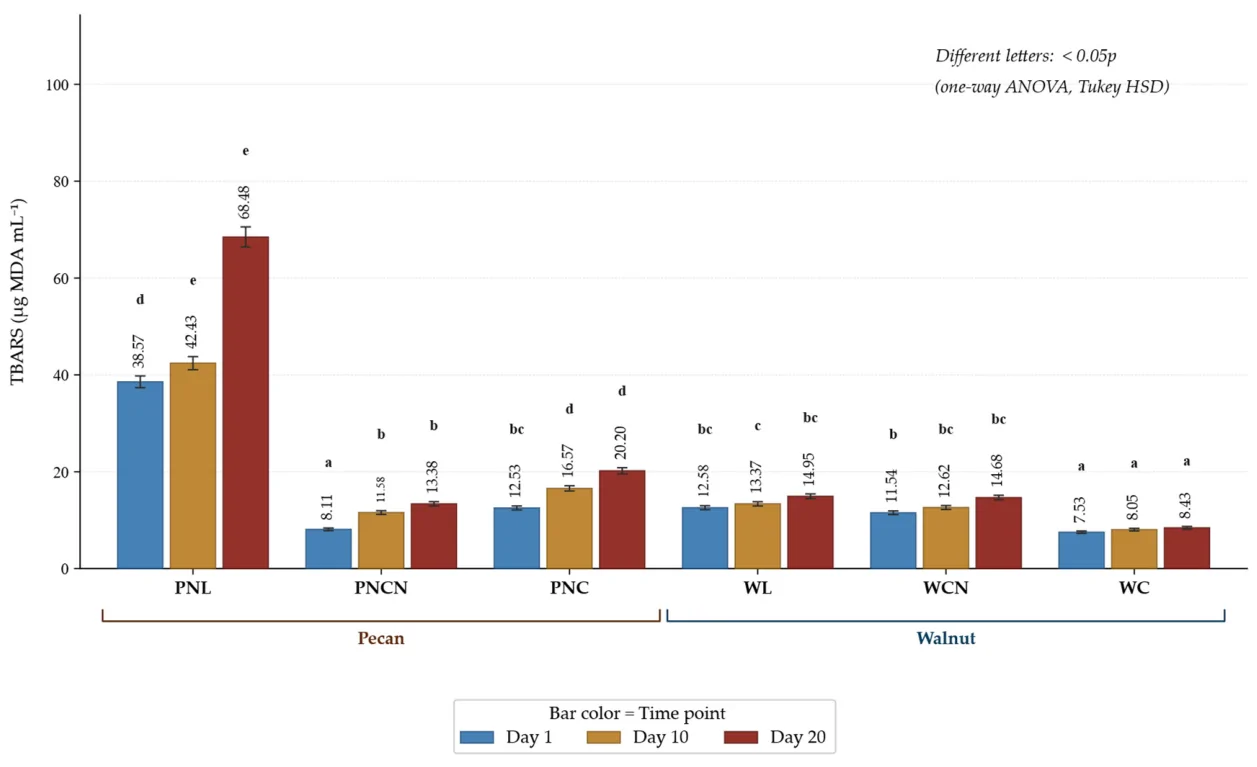
Thiobarbituric acid reactive substances (TBARSs), expressed as μg malondialdehyde (MDA) equivalents/mL for the six composite samples at Days 1, 10, and 20. Values are presented as mean ± SD of three analytical determinations. Different letters indicate differences among the analytical measurements of the six composite samples within each time point (one-way ANOVA followed by Tukey’s HSD test, *p* < 0.05). PNL = pecan, Lovrin; PNCN = pecan, Cluj–Napoca; PNC = pecan, Chisinau; WL = walnut, Lovrin; WCN = walnut, Cluj–Napoca; WC = walnut, Chisinau.

**Figure 2:**
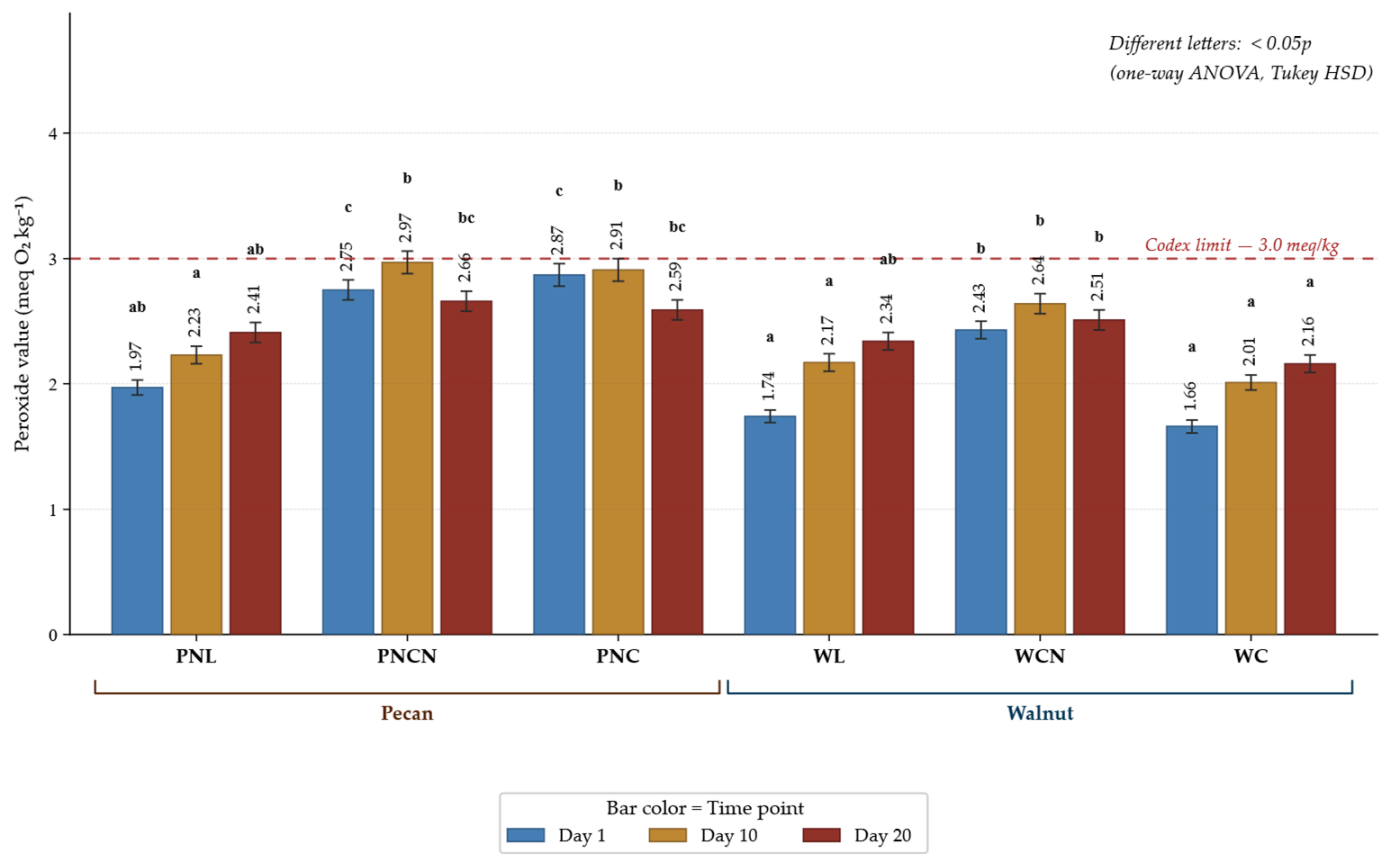
Peroxide value (meq O_2_/kg) for all six sample groups across three time points. Same conventions as Figure 1. Dashed red line marks the Codex Alimentarius recommended limit of 3.0 meq O_2_/kg; no sample exceeded this limit at any time point. Different letters indicate differences among the analytical measurements of the six composite samples within each time point (one-way ANOVA followed by Tukey’s HSD test, *p* < 0.05). PNL = pecan, Lovrin; PNCN = pecan, Cluj–Napoca; PNC = pecan, Chisinau; WL = walnut, Lovrin; WCN = walnut, Cluj–Napoca; WC = walnut, Chisinau.

TBARS and PV data across 20 Days are shown in Figure 1 and Figure 2. Both parameters increased significantly over time in all groups (paired *t*-test, Day 1 vs. Day 20: *p* < 0.001 for TBARS; *p* < 0.05 for PV). All PVs stayed below the 3.0 meq O_2_/kg Codex Alimentarius ceiling throughout.

At the first measurement point (Day 1), TBARS values ranged from 7.5 µg MDA equivalents/mL in WC to 38.6 µg MDA equivalents/mL in PNL. By Day 20, the corresponding values were 8.4 and 68.5 µg MDA equivalents/mL. These results describe differences among the analyzed composite oils but do not identify the chemical factors responsible for those differences. PNCN and PNC showed a biphasic PV pattern: values rose from Day 1 to Day 10, then declined slightly by Day 20 (non-significant, *p* > 0.05). Day 1 TBARS and Day 20 TBARS were near-perfectly correlated across groups (r = +0.994, *p* < 0.001; Table 1). The preservation of the relative oxidative ranking among samples throughout the 20-day storage period is consistent with the findings of Grilo and Wang [18,19], who reported that cultivar-specific TBARS values and volatile oxidation marker profiles in walnut oils remained consistently ranked during 28 weeks of storage under different temperature conditions.

### 2.4. Pearson Correlation Analysis

**Figure 3:**
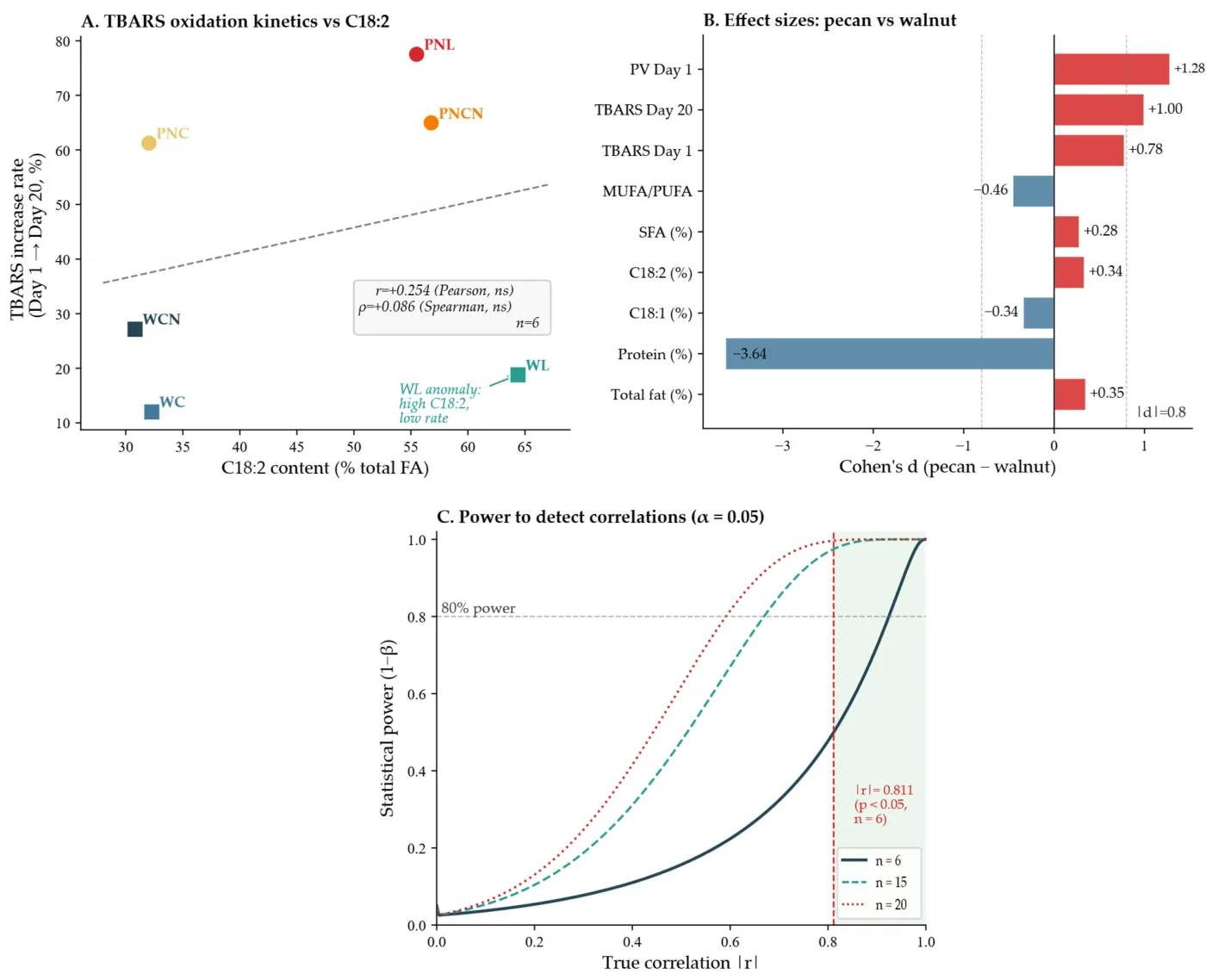
Additional statistical analyses. (**A**) TBARS oxidation rate (Day 1 to Day 20, %) vs. C18:2 content (both correlations non-significant at *n* = 6); the WL anomaly is annotated. (**B**) Exploratory Cohen’s d values for the comparison between the three pecan and walnut composite samples; protein shows the largest observed effect size (d = −3.64). (**C**) Statistical power curves for Pearson correlation detection at alpha = 0.05 for *n* = 6, 15, and 20; vertical line marks the critical r threshold (|r| = 0.811) at *n* = 6, above which significance is achievable. PNL = pecan, Lovrin; PNCN = pecan, Cluj–Napoca; PNC = pecan, Chisinau; WL = walnut, Lovrin; WCN = walnut, Cluj–Napoca; WC = walnut, Chisinau.

Pearson and Spearman correlation coefficients were calculated using the mean values of the six composite samples (*n* = 6); the analytical triplicates were not treated as independent observations. Given the small number of composite samples and the mathematical dependencies among several calculated or compositional variables, these analyses were considered exploratory and descriptive. Complete correlation matrices and selected graphical representations are provided in Tables S1 and S2 and Figures S1 and S2. A reduced comparison of Pearson and Spearman coefficients for selected associations between fatty acid composition and oxidative indicators is retained in Figure 3A, without repeating the corresponding numerical values in the main text.

Additional exploratory analyses are presented in Figure 3A–C. These analyses describe patterns among the six composite-sample means and should not be interpreted as confirmatory evidence of species-level differences or biological mechanisms.

### 2.5. Principal Component Analysis (PCA)

**Figure 4:**
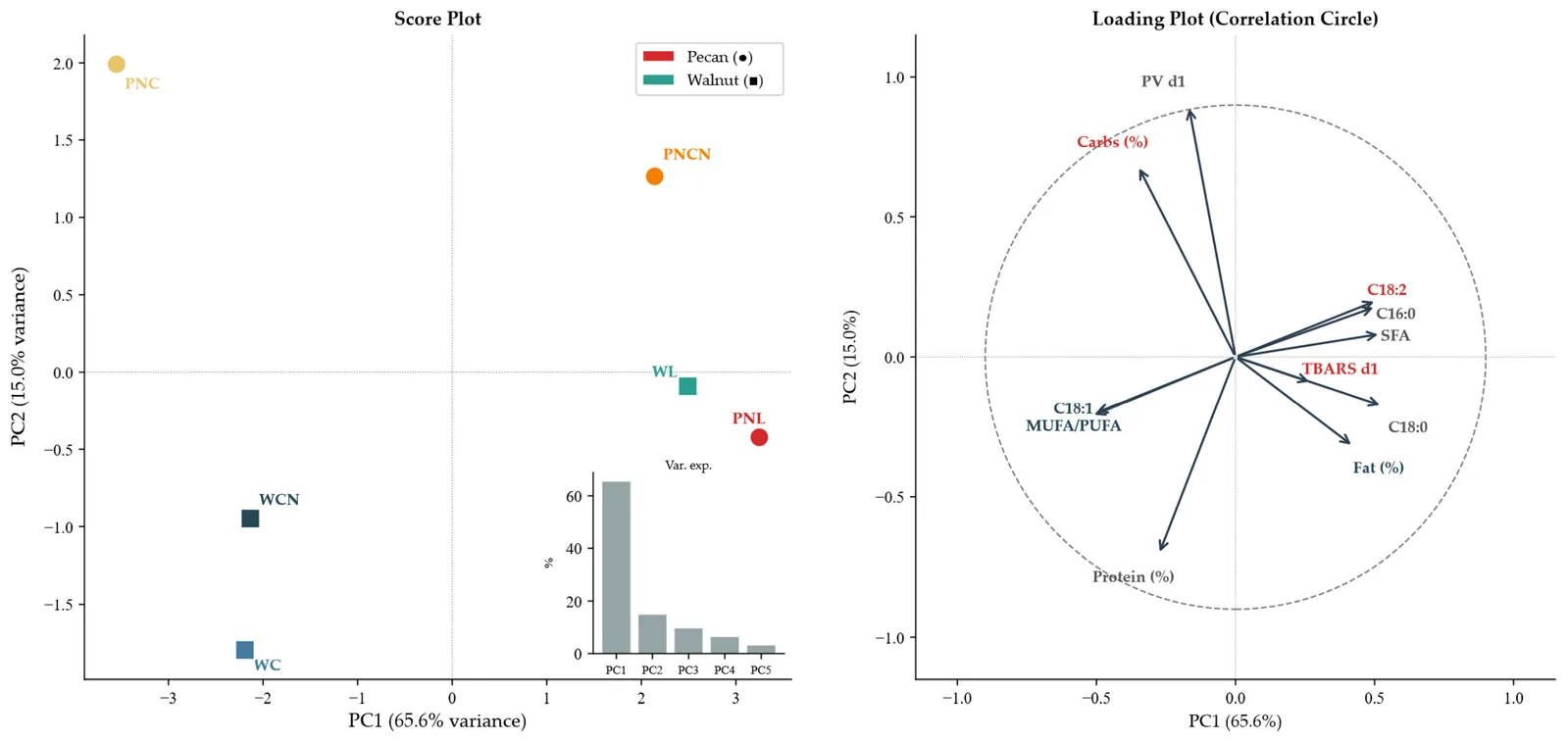
Exploratory PCA biplot of six composite samples based on 11 standardized variables. Left (score plot): PC1 versus PC2 coordinates; the inset bar chart shows the variance explained. Right (correlation circle): variable loadings; arrow direction and length indicate contribution and sign. Red labels indicate PUFA-associated variables, and dark-blue labels indicate MUFA/fat-associated variables. Because the PCA included only six composite samples, sample proximity is descriptive and does not represent confirmatory clustering. PNL = pecan, Lovrin; PNCN = pecan, Cluj–Napoca; PNC = pecan, Chisinau; WL = walnut, Lovrin; WCN = walnut, Cluj–Napoca; WC = walnut, Chisinau.

An exploratory PCA of 11 standardized variables was performed using the mean values of the six composite samples. The first principal component (PC1) and the second principal component (PC2) together summarized 80.6% of the observed variance. However, because the number of observations was smaller than the number of variables, the PCA loadings and sample positions are inherently unstable and may be strongly influenced by individual observations. The PCA shown in Figure 4 is therefore considered only a descriptive visualization and does not establish stable clusters, species-level separation, or biological mechanisms.

## 3. Discussion

### 3.1. Proximate Composition in Context

Among the analyzed composite samples, PNL and PNCN contained 67–68% total fat, whereas the three walnut composite samples contained 59–61%. This sample-level pattern is consistent with values reported in previous studies. Wakeling et al. [6] reported lipid contents of 73.5% and 73.1% for the Australian pecan cultivars Wichita and Western Schley, respectively. Walnut fat values (59.3–61.3%) agree closely with Yerlikaya et al. [30] (61.3–69.4% for Turkish genotypes), Poggetti et al. [23] (54.2–72.2% for accessions from north-eastern Italy), Trandafir et al. [26] (59.0–66.2% for Romanian cultivars), and Rebufa et al. [3] comprehensive review (52–74% across multiple regions).

The Chisinau pecan composite sample (PNC) had a lower total fat value (51.9%) than PNL and PNCN. Similar variation in total fat content among pecan accessions has been reported in previous studies [9,34]. However, cultivar identity, genotype, soil characteristics, nutrient availability, and climatic parameters were not determined in the present study. Therefore, the lower total fat value observed in PNC cannot be attributed to a specific genetic or environmental factor. The strong association between total fat and calculated energy value was expected because energy was derived from fat, protein, and carbohydrate contents using Atwater factors. Similarly, carbohydrate content was calculated by difference and was therefore mathematically dependent on the measured proximate components. These correlations were not interpreted as independent biological relationships.

### 3.2. Fatty Acid Profile: Interpretation and Practical Context

Across the six composite-sample means, C18:1 and C18:2 showed a strong inverse association. Because these variables were expressed as percentages of the same total, the strength of this association is influenced by the compositional structure of the data and should not be interpreted as evidence of a direct biological mechanism. Previous studies have identified the Δ12–fatty acid desaturase FAD2 as the enzyme involved in the conversion of oleic acid to linoleic acid [20,21,34,35]. However, FAD2 expression, genotype, cultivar identity, phenological observations, meteorological data corresponding to kernel development and harvest, and site-specific soil characteristics were not available in the present study. Consequently, the observed differences in fatty acid composition cannot be attributed to climatic or soil conditions, and the present study does not evaluate environmental regulation of FAD2 activity. The FAD2 pathway is mentioned only as biological context derived from previous literature and not as a mechanism demonstrated by the present results. Associations between fatty acid variables and TBARS were not statistically significant.

Among the analyzed composite samples, WL contained 64.4% C18:2 and 22.5% C18:1, while its TBARS increase from Day 1 to Day 20 was 1.19-fold, compared with 1.27-fold in WCN and 1.78-fold in PNL. Low C18:1 values have also been reported previously in walnut accessions [26]. However, the genotype, cultivar identity, and antioxidant composition of WL were not determined; therefore, the factors responsible for its fatty acid profile and oxidative behavior cannot be established. PNC showed a relative fatty acid profile similar to those of WCN and WC, with 56.8% C18:1 and 32.0% C18:2. Because genotype, cultivar identity, harvest-year conditions, and environmental parameters were not determined, the reason for this similarity cannot be established from the present study.

α-linolenic acid (C18:3n–3) contains three double bonds and is therefore more susceptible to oxidation than oleic and linoleic acids [3]. Variations in its relative proportion may consequently affect the oxidative behavior of oils. In the present study, however, α-linolenic acid was detected only at trace proportions in WL (0.16%) and PNL (0.02%) and was not detected in the other four composite oils. Therefore, its specific contribution to the observed differences in PV and TBARS cannot be determined from the present data.

### 3.3. Oxidative Stability: Mechanisms and Food Industry Implications

Mechanical hot pressing at 120 ± 2 °C can increase oil recovery, but exposure to elevated temperature may also accelerate lipid oxidation, promote hydroperoxide decomposition, and reduce heat-sensitive antioxidant constituents [10]. The higher TBARS values observed in PNL than in WC were already present at the first measurement point. However, because the study did not include unpressed material or a cold-pressed control, the specific contribution of hot pressing cannot be distinguished from the effects of initial sample composition, subsequent handling, or storage. Previous research comparing raw and roasted pecans has likewise shown that thermal processing can influence oxidative stability during storage [36]. Oxidative behavior is influenced not only by fatty acid composition but also by tocopherols, phenolic compounds, phytosterols, and other antioxidant and pro-oxidant constituents. Pei et al. [37] also showed that antioxidant type and combinations affected the oxidative stability of walnut oil under accelerated storage conditions. Previous research has shown that tocopherol and phytosterol concentrations in pecan oil can vary among growing locations [38]. These compounds were not measured in the present study. Consequently, the differences in PV and TBARS cannot be attributed solely to fatty acid composition.

Tocopherol content is the second major determinant of oxidative stability in nut oils. Tsao et al. [39] demonstrated that pecan oil, owing to its high gamma-tocopherol content (52.8–60.3 mg/100 g), maintains oxidative stability comparable to peanut oil despite similar PUFA levels. Pycia et al. [40] showed that walnut kernel tocopherols vary markedly with cultivar and maturity stage (1.76–18.30 mg/100 g total), directly modulating oxidative stability. We did not measure tocopherols, an acknowledged limitation. Zhang et al. [41] reviewed PV analytical methods and confirmed that iodometric titration ([22], as used in this study) remains the regulatory reference standard for values in the 0–10 meq O_2_/kg range.

The biphasic PV trajectory in PNCN and PNC (rise from Day 1 to Day 10, slight decline by Day 20) may be consistent with classical two-phase lipid oxidation kinetics: hydroperoxide accumulation peaks, then decomposition to secondary products exceeds formation [3,42]. Elouafy et al. [43] documented the same biphasic pattern for roasted walnut oil. All PVs remained below 3.0 meq O_2_/kg throughout the study, confirming compliance with the Codex Alimentarius criterion. Gao et al. [44] showed that walnut oil produced by different processing methods retained its cholesterol-lowering bioactivity regardless of PV level, suggesting that functional quality depends on the full phytochemical profile, not PV alone. The first analytical time point was Day 1, after decantation and filtration, and no separate measurement immediately after pressing was obtained. Therefore, oxidative changes occurring before the first measurement cannot be distinguished from those occurring during subsequent storage.

### 3.4. PCA and Multivariate Interpretation

Principal component analysis is widely used as an exploratory multivariate method for summarizing variance patterns in complex datasets [45]. The exploratory PCA of 11 standardized variables explained 80.6% of the total variance in the first two components (PC1: 65.6%; PC2: 15.0%). PC1 primarily contrasted C18:2, TBARS Day 1, and SFA loadings on its positive side with C18:1, the MUFA/PUFA ratio, and total fat loadings on its negative side. PC2 contrasted carbohydrate content and initial PV with protein and fat content. In the score plot, PNL and WL were positioned on the positive side of PC1, whereas WCN, WC, and PNC were positioned on its negative side. PNC appeared closer to WCN and WC than to the other pecan samples. These positions describe only the multivariate structure of the six analyzed composite samples. Because the PCA was based on only six composite samples, the loadings and sample positions may be strongly influenced by individual observations. Therefore, the analysis does not establish stable sample clusters, confirm species-level separation, or demonstrate biochemical mechanisms. The PCA should be regarded only as an exploratory visualization of the present dataset, and its patterns require confirmation using a larger number of independent biological samples. Protein loads strongly on the negative PC2 pole, orthogonal to the fatty acid axes, indicating that variation in protein values among the six composite samples is oriented independently of the fatty acid variables in this exploratory analysis. PV Day 1 loads on positive PC2, partially orthogonal to the main fatty acid axis, consistent with the weak and non-significant correlations between PV and individual fatty acids reported in Table 3.

## 4. Materials and Methods

### 4.1. Plant Material and Experimental Design

**Figure 5:**
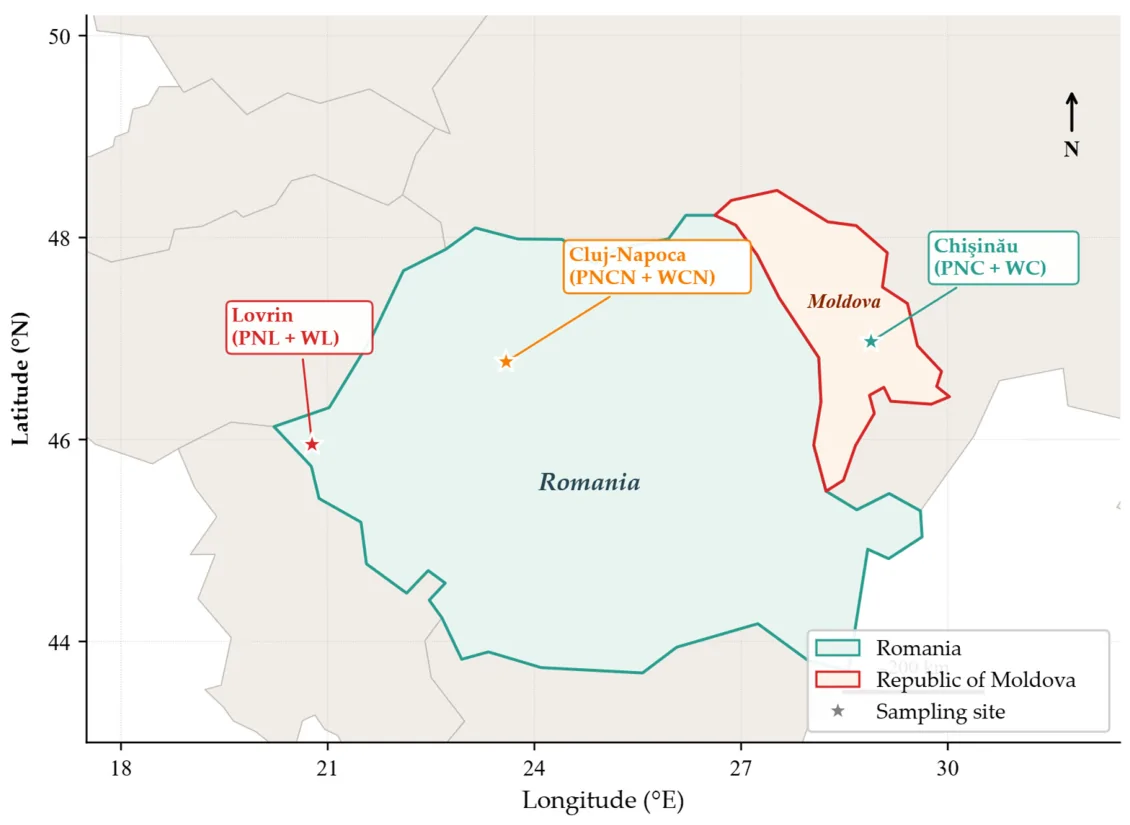
Geographical location of the three sampling sites in Romania (Lovrin and Cluj–Napoca) and the Republic of Moldova (Chisinau). Stars mark each site; each contributed one pecan and one walnut sample group.

*Carya illinoinensis* and *Juglans regia* L. kernels were harvested at full physiological maturity in autumn 2022 and 2023 from three sites (Figure 5): Lovrin, Timis County, Romania (45°57′ N, 20°46′ E); Cluj–Napoca, Cluj County, Romania (46°46′ N, 23°35′ E); and the Botanical Garden of the National Institute Alexandru Ciubotaru, Chisinau, Republic of Moldova (46°58′ N, 28°52′ E). At each site and for each species, approximately 5 kg of nuts were collected from the ground beneath at least five mature trees (approximately 30 years old). Natural abscission, dehiscence of the hull in walnut or of the shuck in pecan, and the presence of a fully developed, hardened shell were used as field indicators of full physiological maturity. Only intact nuts without visible physical damage or signs of fungal contamination were retained for analysis. Because the nuts were collected from the ground after natural abscission, their original position within the tree canopy could not be determined. The collected material was transported to the laboratory in insulated containers. The position of individual nuts within the tree canopy was [recorded/not recorded]. The collected material was transported to the laboratory in insulated containers. No pesticides, fertilizers, or irrigation were applied. Six composite samples were defined: PNL (pecan, Lovrin), PNCN (pecan, Cluj–Napoca), PNC (pecan, Chisinau), WL (walnut, Lovrin), WCN (walnut, Cluj–Napoca), and WC (walnut, Chisinau). For each site × species combination, kernels collected from at least five trees were pooled into a single composite sample. Therefore, the biological experimental unit was the composite site × species sample. Each laboratory determination was performed in triplicate using aliquots from the same composite sample; these measurements represent analytical replicates and not independent biological replicates. Consequently, the analytical triplicates were used to estimate analytical repeatability, whereas the six composite-sample means were used for exploratory comparisons. The experimental design does not allow population-level inference regarding geographical location, environmental effects, or species differences.

### 4.2. Oil Extraction by Hot Pressing

Kernels were dried at 60 +/− 1 °C for 120 min in a forced-air oven to residual moisture below 5% (*w*/*w*), verified by AOAC 925.40–1925. Oil was extracted using a mechanical expeller press (OMac PU 400, O’MAC S.R.L., Arges, Romania) at 120 ± 2 °C; hot-press temperatures in the 100–130 °C range maximize yield relative to cold pressing [35,46,47]. Raw oil was decanted at 20 ± 2 °C for 24 h, filtered through qualitative filter paper, and stored at 4 °C in dark glass vials. Extraction yield (%) = (moil/mdry kernel) × 100 [48].

### 4.3. Proximate Composition of Kernels

Proximate composition was determined on whole kernels for all six groups (Analytical Reports 35C/28 March 2024, University of Life Sciences King Mihai I from Timisoara). Moisture: oven-drying at 103 +/− 2 °C to constant weight (AOAC 925.40–1925). Ash: muffle incineration at 550 °C for 6 h (AOAC 950.49–1950). Crude protein: Kjeldahl digestion (AOAC 950.48), conversion factor N × 5.30 [49]. Total lipids: Soxhlet extraction with hexane (AOAC 948.22–1948). Carbohydrates: calculated by difference. Caloric value: Atwater factors (37 kJ/g lipids; 17 kJ/g protein and carbohydrates).

### 4.4. Fatty Acid Composition by GC–MS

Fatty acid methyl esters (FAMEs) were prepared from 0.1 g of each extracted oil according to AOAC 969.33 and previously described procedures [50,51]. The oil sample was treated with 3 mL of a 20% boron trifluoride solution in methanol. Derivatization was performed for 1 h at 80 °C in an ultrasonic bath. After cooling, 2.5 mL of 10% sodium chloride solution was added, and the FAMEs were extracted into 2 mL of hexane. The organic phase was separated by centrifugation at 3000 rpm for 15 min, and 1 µL of the hexane fraction was injected into the GC–MS system.

GC–MS analyses were performed using a GCMS–QP2010 Plus system (Shimadzu, Kyoto, Japan) equipped with an AT–WAX capillary column (30 m × 0.32 mm i.d. × 1.0 μm film thickness). Helium was used as the carrier gas at a flow rate of 1.0 mL/min and a linear velocity of 37.8 cm/s. The oven temperature was maintained at 140 °C for 10 min, increased at 7 °C/min to 250 °C, and then maintained at 250 °C for 10 min. The total analysis time was 35.71 min. The injection was performed using a split ratio of 1:10. The injector, ion-source, and GC–MS interface temperatures were 250, 210, and 255 °C, respectively.

FAMEs were identified using two concordant criteria: (i) comparison of their mass spectra with those included in the National Institute of Standards and Technology 2005 (NIST05) mass-spectral library and (ii) comparison of their retention times with those of the corresponding compounds in a Supelco 37-component FAME reference mixture analyzed under the same chromatographic conditions. Peak assignments were accepted only when the NIST05 library identification agreed with the corresponding FAME indicated by the reference mixture. Compound identification was therefore not based exclusively on mass-spectral library matching. The archived analytical records do not contain the individual NIST05 similarity scores or a documented numerical mass-spectrum matching-score threshold; consequently, these values cannot be reported retrospectively. The Supelco mixture was used for compound identification and not as an internal quantitative standard.

No internal standard or multipoint quantitative calibration was used because the analysis was designed to determine relative fatty acid composition rather than absolute fatty acid concentrations. Relative fatty acid composition was calculated by peak area normalization according to the following equation:

Relative fatty acid (%) = (Ai/ΣA) × 100,

where Ai is the peak area of an individual identified FAME and ΣA is the total peak area of all identified FAMEs. Therefore, the results are reported as percentages of total identified FAME peak area and not as absolute fatty acid concentrations. Each composite oil sample was analyzed in analytical triplicate, and analytical repeatability was evaluated from the standard deviation of the three measurements.

### 4.5. TBARS Determination

TBARS were measured spectrophotometrically at lambda = 532 nm, a standard colorimetric assay for secondary lipid oxidation products in vegetable oils [52], against a malondialdehyde (MDA, 1,1,3,3–tetraethoxypropane, Sigma–Aldrich, St. Louis, MO, USA) calibration curve (0–5 μm/L). Briefly, 0.5 mL of oil was mixed with 2.5 mL 20% trichloroacetic acid (TCA) (*w*/*v*) and 2.5 mL 0.67% thiobarbituric acid (TBA) (*w*/*v*), incubated at 95 °s C for 30 min, centrifuged at 3000× *g* for 10 min, and the supernatant read at 532 nm (Specord 205, Analytik Jena, Jena, Germany). Results were expressed as μg MDA equivalents/mL. Following hot pressing, the oils were decanted for 24 h and filtered. The first TBARS determination was performed immediately after filtration and was designated Day 1; this represented the first available analytical time point. No separate measurement immediately after pressing (Day 0) was performed. Subsequent determinations were performed on Days 10 and 20, with the samples stored at 4 °C in darkness between measurements.

### 4.6. Peroxide Value

PV was determined by iodometric titration following SR EN ISO 3960:2017. Oil (1.00 +/− 0.01 g) was dissolved in chloroform:glacial acetic acid, treated with saturated KI solution for 1 min in darkness, diluted with degassed water, and titrated against 0.01 mol/L Na_2_S_2_O_3_ using starch indicator: PV (meq O_2_/kg) = [(Vs − Vb) × c × 1000]/m. The Codex Alimentarius ceiling for fresh edible vegetable oils is 3.0 meq O_2_/kg (Stan 210–1999). Measurements at Days 1, 10, and 20 [43].

### 4.7. Statistical Analysis

All determinations were performed in analytical triplicate, and data are reported as mean ± SD. Normality was assessed using the Shapiro–Wilk test, and homogeneity of variance was evaluated using Levene’s test. One-way ANOVA followed by Tukey’s HSD test was used to compare the analytical measurements obtained for the six composite samples (α = 0.05). Because the triplicates were analytical rather than biological replicates, the resulting *p*-values and superscript letters describe differences among the specific composite samples relative to analytical variability and cannot be generalized to populations, species, cultivars, or geographical locations. Pearson (r) and Spearman rank (ρ) correlation coefficients were calculated using the mean value of each composite sample (*n* = 6) and were interpreted as exploratory. Because the fatty acid variables were expressed as percentages of a common total, both coefficients may be influenced by the compositional structure of the data and were not interpreted as evidence of direct biological mechanisms. PCA was performed on z-score-standardized mean values of the six composite samples and was interpreted as an exploratory analysis rather than as confirmatory evidence of sample clustering.

## 5. Conclusions

Hot-pressed pecan and walnut oils from Romania and Moldova met fresh edible-oil quality standards, with all peroxide values below 3.0 meq O_2_/kg for 20 days, but diverged in ways that matter for storage and food applications.

PNL and PNCN were fat-rich (67–68%) and linoleic-dominant (55–57% C18:2n–6), appealing as n–6 dietary sources but showed higher TBARS values than some walnut oils under the storage conditions tested. WC, the most oleic-dominant sample, had TBARS values five to eight times lower than PNL at Day 20. TBARS values differed among the analyzed composite oils; however, these differences cannot be attributed solely to fatty acid composition because antioxidant constituents were not measured. In addition, correlations between fatty acid variables and TBARS were not statistically significant.

PNC contained a higher relative proportion of C18:1 and a lower proportion of C18:2 than the other two pecan composite samples. Although these proportions were similar to those observed in WCN and WC, the factors responsible for this sample-level similarity cannot be determined because cultivar identity, genotype, and environmental parameters were not available.

Across the six composite-sample means, C18:1 and C18:2 showed a strong inverse association. Because both variables were expressed as percentages of the same total, this relationship may be strongly influenced by compositional closure and should be interpreted as descriptive rather than mechanistic.

These findings provide a preliminary regional baseline for future genotype-resolved studies and oil-quality evaluation. However, the present sampling design does not support specific cultivar-selection or industrial-processing recommendations.

## Figures and Tables

**Table 1:** Proximate composition of pecan and walnut kernels from three growing sites (mean +/− SD, *n* = 3). Different superscript letters within a column indicate significant differences (ANOVA, Tukey HSD, *p* < 0.05). SFA = saturated fatty acids by GC–MS.

Sample	Moisture (%)	Ash (%)	Crude Protein (%)	Total Fat AOAC (%)	SFA GC–MS (%)	Carbohydrates (%)	Energy (kcal/100 g)
PNL	2.55 ± 0.04 ^ab^	1.62 ± 0.03 ^a^	9.00 ± 0.18 ^a^	68.21 ± 1.21 ^c^	6.59 ± 0.12 ^b^	18.62 ± 0.54 ^a^	724.37 ± 9.1 ^c^
PNCN	2.36 ± 0.05 ^a^	1.32 ± 0.04 ^a^	8.43 ± 0.22 ^a^	67.42 ± 1.05 ^c^	6.06 ± 0.10 ^a^	20.46 ± 0.61 ^a^	722.36 ± 8.7 ^c^
PNC	3.99 ± 0.08 c	2.63 ± 0.06 ^b^	10.29 ± 0.31 ^b^	51.89 ± 0.98 ^a^	4.58 ± 0.09 ^a^	31.20 ± 0.88 ^b^	632.94 ± 7.4 ^a^
WL	2.57 ± 0.06 ^ab^	1.90 ± 0.04 ^ab^	12.19 ± 0.28 ^c^	59.94 ± 1.14 ^b^	6.59 ± 0.11 ^b^	23.40 ± 0.72 ^c^	681.81 ± 8.2 ^b^
WCN	2.17 ± 0.04 ^a^	1.99 ± 0.05 ^ab^	14.42 ± 0.35 ^d^	59.33 ± 1.08 ^b^	6.75 ± 0.13 ^b^	22.09 ± 0.67 ^c^	680.04 ± 8.0 ^b^
WC	2.93 ± 0.07 ^bc^	1.64 ± 0.04 ^a^	12.72 ± 0.29 ^c^	61.34 ± 1.17 ^b^	6.88 ± 0.12 ^b^	21.37 ± 0.65 ^c^	688.45 ± 8.5 ^b^

PNL = pecan Lovrin; PNCN = pecan Cluj–Napoca; PNC = pecan Chisinau; WL = walnut Lovrin; WCN = walnut Cluj–Napoca; WC = walnut Chisinau. SFA = saturated fatty acids by GC–MS. Different letters within a column indicate significant differences (one-way ANOVA, Tukey’s honestly significant difference (HSD) test, *p* < 0.05). Values are mean ± standard deviation (SD) of three analytical determinations. Different superscript letters indicate differences among the analytical measurements of the six composite samples. These comparisons apply only to the analyzed composite samples and do not represent biological variability among trees, populations, species, or geographical locations.

**Table 2:** Relative fatty acid composition determined by GC–MS and expressed as percentage of total identified fatty acid methyl ester (FAME) peak area. Values are mean ± SD of three analytical determinations.

FA	Common Name	PNL	PNCN	PNC	WL	WCN	WC
C16:1n–7 (∆9)	*Palmitoleic acid*	0.038 ± 0.002 ^b^	0.030 ± 0.001 ^ab^	0.024 ± 0.001 ^a^	0.031 ± 0.001 ^ab^	0.010 ± 0.001 ^a^	0.010 ± 0.001 ^a^
C16:1n–9 (∆7)	*Hypogeic acid*	0.061 ± 0.003 ^b^	0.073 ± 0.003 ^b^	0.091 ± 0.004 ^c^	0.062 ± 0.003 ^b^	0.029 ± 0.001 ^a^	0.029 ± 0.001 ^a^
C16:0	*Palmitic acid*	7.83 ± 0.19 ^b^	7.88 ± 0.21 ^b^	6.53 ± 0.17 ^a^	8.10 ± 0.22 ^b^	7.00 ± 0.19 ^ab^	6.38 ± 0.16 ^a^
C17:0	*Margaric acid*	0.026 ± 0.001 ^a^	0.029 ± 0.001 ^a^	0.031 ± 0.001 ^a^	0.034 ± 0.001 ^a^	0.036 ± 0.001 ^a^	0.031 ± 0.001 ^a^
C18:2n–6 (∆9,12)	*Linoleic acid*	55.50 ± 1.24 ^c^	56.78 ± 1.31 ^c^	32.03 ± 0.88 ^a^	64.39 ± 1.42 ^d^	30.81 ± 0.85 ^a^	32.28 ± 0.89 ^a^
C18:1n–9 (∆9)	*Oleic acid*	31.52 ± 0.78 ^b^	30.87 ± 0.74 ^b^	56.78 ± 1.33 ^c^	22.48 ± 0.61 ^a^	57.86 ± 1.35 ^c^	57.12 ± 1.32 ^c^
C18:1 ∆12	*Oleic acid (D12)*	1.435 ± 0.04 ^b^	1.116 ± 0.03 ^a^	2.011 ± 0.05 ^c^	1.246 ± 0.03 ^ab^	1.397 ± 0.04 ^ab^	1.355 ± 0.04 ^ab^
C18:0	*Stearic acid*	3.376 ± 0.09 ^b^	3.109 ± 0.08 ^b^	2.285 ± 0.06 ^a^	3.280 ± 0.09 b	2.671 ± 0.07 ^a^	2.617 ± 0.07 ^a^
C18:3n–3 (∆9,12,15)	*α–linolenic acid (ALA)*	0.019 ± 0.001 ^a^	nd	nd	0.159 ± 0.005 ^b^	nd	nd
C20:1	*Eicosenoic acid*	0.130 ± 0.004 ^b^	0.069 ± 0.002 ^a^	0.128 ± 0.004 ^b^	0.152 ± 0.005 ^b^	0.106 ± 0.003 ^ab^	0.113 ± 0.003 ^ab^
C20:0	*Arachidic acid*	0.066 ± 0.002 ^b^	0.053 ± 0.002 ^a^	0.088 ± 0.003 ^c^	0.064 ± 0.002 ^b^	0.068 ± 0.002 ^b^	0.059 ± 0.002 ^ab^

FA = fatty acid; nd = not detected. Different superscript letters within a row indicate significant differences (one-way ANOVA, Tukey HSD, *p* < 0.05). Values are mean ± SD of three analytical determinations. Different superscript letters indicate differences among the analytical measurements of the six composite samples. These comparisons apply only to the analyzed composite samples and do not represent biological variability among trees, populations, species, or geographical locations.

**Table 3:** Spearman rank correlations (rho) compared to Pearson r for key variable pairs (*n* = 6 sample groups). *** *p* < 0.001; ** *p* < 0.01; ns = not significant. See footnote for compositional data constraint caveat.

Variable Pair	Pearson r	Pearson p	Spearman Rho	Spearman p	Concordant?
C18:1 vs. C18:2	−0.9998	<0.001 ***	−0.9429	0.005 **	Partial (1)
Fat vs. Energy	+0.9980	<0.001 ***	+1.0000	<0.001 ***	Yes
Fat vs. Carbs	−0.9256	0.008 **	−0.9429	0.005 **	Yes
TBARS d1 vs. d20	+0.9938	<0.001 ***	+0.9429	0.005 **	Yes
PV d1 vs. d20	+0.9329	0.007 **	+0.9429	0.005 **	Yes
C18:2 vs. TBARS d1	+0.332	0.520 ns	+0.257	0.623 ns	Yes
MUFA/PUFA vs. TBARS d1	−0.379	0.458 ns	−0.257	0.623 ns	Yes
C18:2 vs. TBARS rate (%)	+0.254	0.627 ns	+0.086	0.872 ns	Yes

(1) Both Pearson and Spearman coefficients are based on relative percentage data and remain affected by the closed compositional structure. Therefore, the C18:1–C18:2 association is interpreted as exploratory and does not confirm a direct biosynthetic mechanism.

## Data Availability

Data Availability Statement: Analytical reports (Nos. 35C/28.03.2024 and 16C/25.03.2024) and the numerical results supporting the findings of this study are held at the Interdisciplinary Research Platform (PCI), University of Life Sciences King Mihai I, Timisoara, and are available from the corresponding authors upon reasonable request. The original GC–MS chromatographic files and individual NIST05 library-search reports could not be recovered from the laboratory archive.

## Supplementary Materials

The following supporting information can be downloaded at: https://www.mdpi.com/article/10.3390/molecules31173093/s1. Figure S1: Pearson correlation heatmap; Figure S2: Selected bivariate scatter plots; Table S1: Pearson correlation matrix for fatty acid variables; Table S2: Pearson correlation matrix for proximate composition, fatty acids, and oxidative indicators.

## Abbreviations

ANOVA, analysis of variance; FA, fatty acid; FAME, fatty acid methyl ester; GC–MS, gas chromatography–mass spectrometry; MDA, malondialdehyde; MUFAs, monounsaturated fatty acids; PNC, pecan nut, Chisinau; PNCN, pecan nut, Cluj–Napoca; PNL, pecan nut, Lovrin; PV, peroxide value; PUFAs, polyunsaturated fatty acids; SD, standard deviation; SFAs, saturated fatty acids; TBARSs, thiobarbituric acid reactive substances; WC, walnut, Chisinau; WCN, walnut, Cluj–Napoca; WL, walnut, Lovrin.
