# Supplementary material for: Fatty Acid Composition and Oxidative Changes in Pecan and Walnut Oils from Eastern Europe
Source: Molecules. 2026 Sep 3;31(17):3093. doi: 10.3390/molecules31173093 (PMC13567129; doi:10.3390/molecules31173093)
Supplement: Supplementary file 1 [file molecules-31-03093-s001.zip › molecules-4494845-supplementary.pdf]

Table S1. Pearson correlation matrix for major fatty acid variables (n = 6 sample groups). *** p < 0.001; ** p < 0.01; * p < 0.05. SFA = saturated FA; MUFA = monounsaturated FA; PUFA = polyunsaturated FA.

	**C16:0**	**C18:0**	**C18:1**	**C18:2**	**SFA**	**MUFA**	**PUFA**
C16:0	1	+0.922**	–0.954**	+0.950**	+0.990***	–0.954**	+0.950**
C18:0	+0.922**	1	–0.910*	+0.908*	+0.967**	–0.910*	+0.908*
C18:1	–0.954**	–0.910*	1	–1.000***	–0.956**	+1.000***	–1.000***
C18:2	+0.950**	+0.908*	–1.000***	1	+0.952**	–1.000***	+1.000***
SFA	+0.990***	+0.967**	–0.956**	+0.952**	1	–0.956**	+0.952**
MUFA	–0.954**	–0.910*	+1.000***	–1.000***	–0.956**	1	–1.000***
PUFA	+0.950**	+0.908*	–1.000***	+1.000***	+0.952**	–1.000***	1

**** p < 0.001; ** p < 0.01; * p < 0.05. SFA = saturated FA; MUFA = monounsaturated FA; PUFA = polyunsaturated FA.*

Table S2. Pearson correlation matrix for proximate composition, major fatty acids, and oxidative stability indicators (n = 6 sample groups). *** p < 0.001; ** p < 0.01; * p < 0.05; ns = not significant.

	**Fat**	**Protein**	**Energy**	**Carbs**	**C18:1**	**C18:2**	**MUFA/P**	**TBARS d20**	**PV d20**
Fat	1	–0.448ns	+0.998***	–0.926**	–0.586ns	+0.588ns	–0.638ns	+0.459ns	–0.053ns
Protein	–0.448ns	1	–0.400ns	+0.082ns	+0.507ns	–0.508ns	+0.578ns	–0.490ns	–0.464ns
Energy	+0.998***	–0.400ns	1	–0.941**	–0.581ns	+0.583ns	–0.629ns	+0.421ns	–0.061ns
Carbs	–0.926**	+0.082ns	–0.941**	1	+0.420ns	–0.421ns	+0.451ns	–0.344ns	+0.291ns
C18:1	–0.586ns	+0.507ns	–0.581ns	+0.420ns	1	–1.000***	+0.995***	–0.335ns	–0.076ns
C18:2	+0.588ns	–0.508ns	+0.583ns	–0.421ns	–1.000***	1	–0.995***	+0.321ns	+0.069ns
MUFA/P	–0.638ns	+0.578ns	–0.629ns	+0.451ns	+0.995***	–0.995***	1	–0.380ns	–0.098ns
TBARS d20	+0.459ns	–0.490ns	+0.421ns	–0.344ns	–0.335ns	+0.321ns	–0.380ns	1	+0.019ns
PV d20	–0.053ns	–0.464ns	–0.061ns	+0.291ns	–0.076ns	+0.069ns	–0.098ns	+0.019ns	1

**** p < 0.001; ** p < 0.01; * p < 0.05; ns = not significant. MUFA/P = MUFA/PUFA ratio; n = 6 sample groups.*

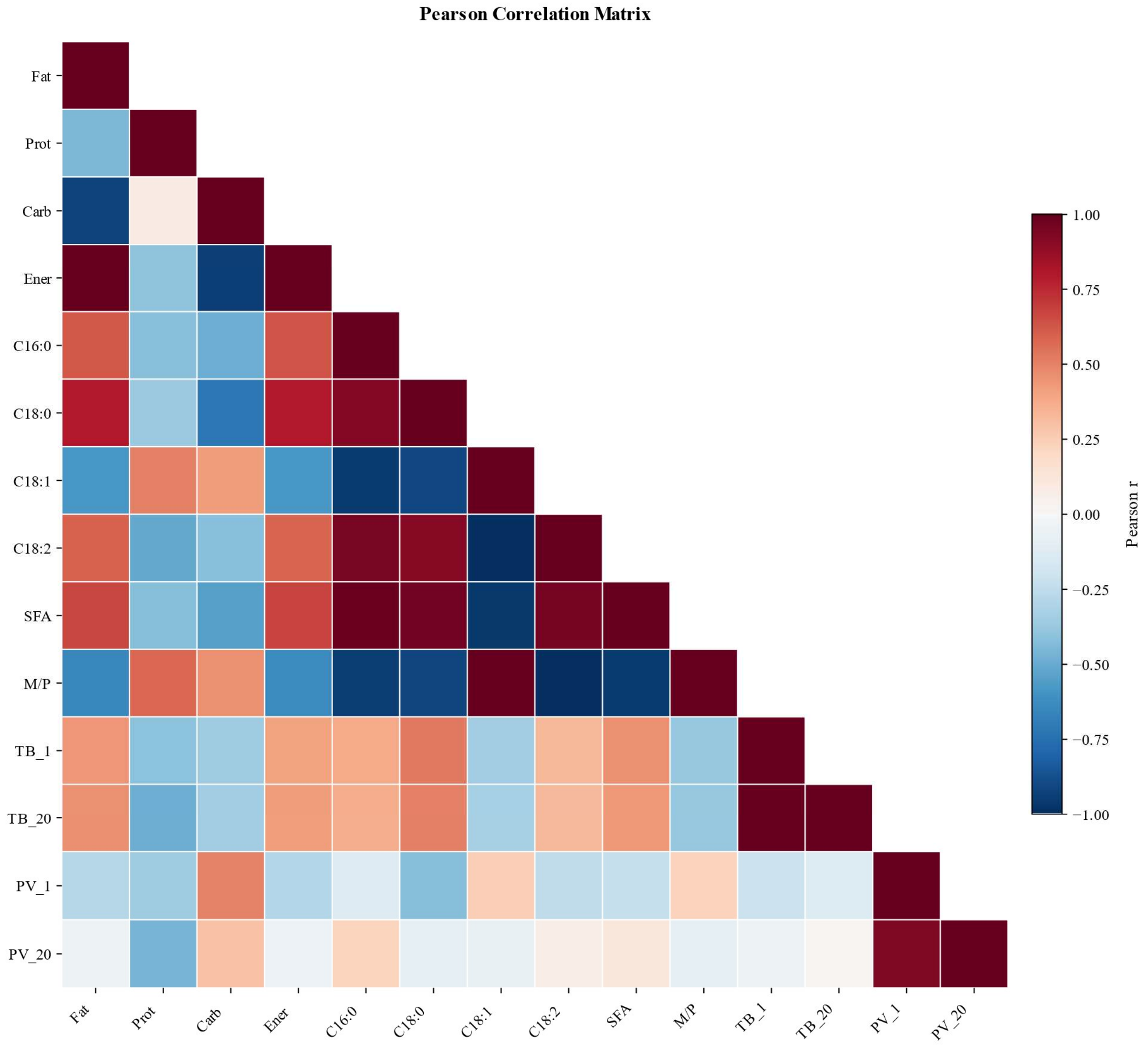

Figure S1. Pearson correlation heatmap for all 14 measured variables (n = 6 sample groups). Lower triangle shown; diagonal = 1.00. Red = positive correlation; blue = negative. Values shown as r with significance stars. Variables grouped by analytical block: Proximate, Fatty acids, Oxidative stability.

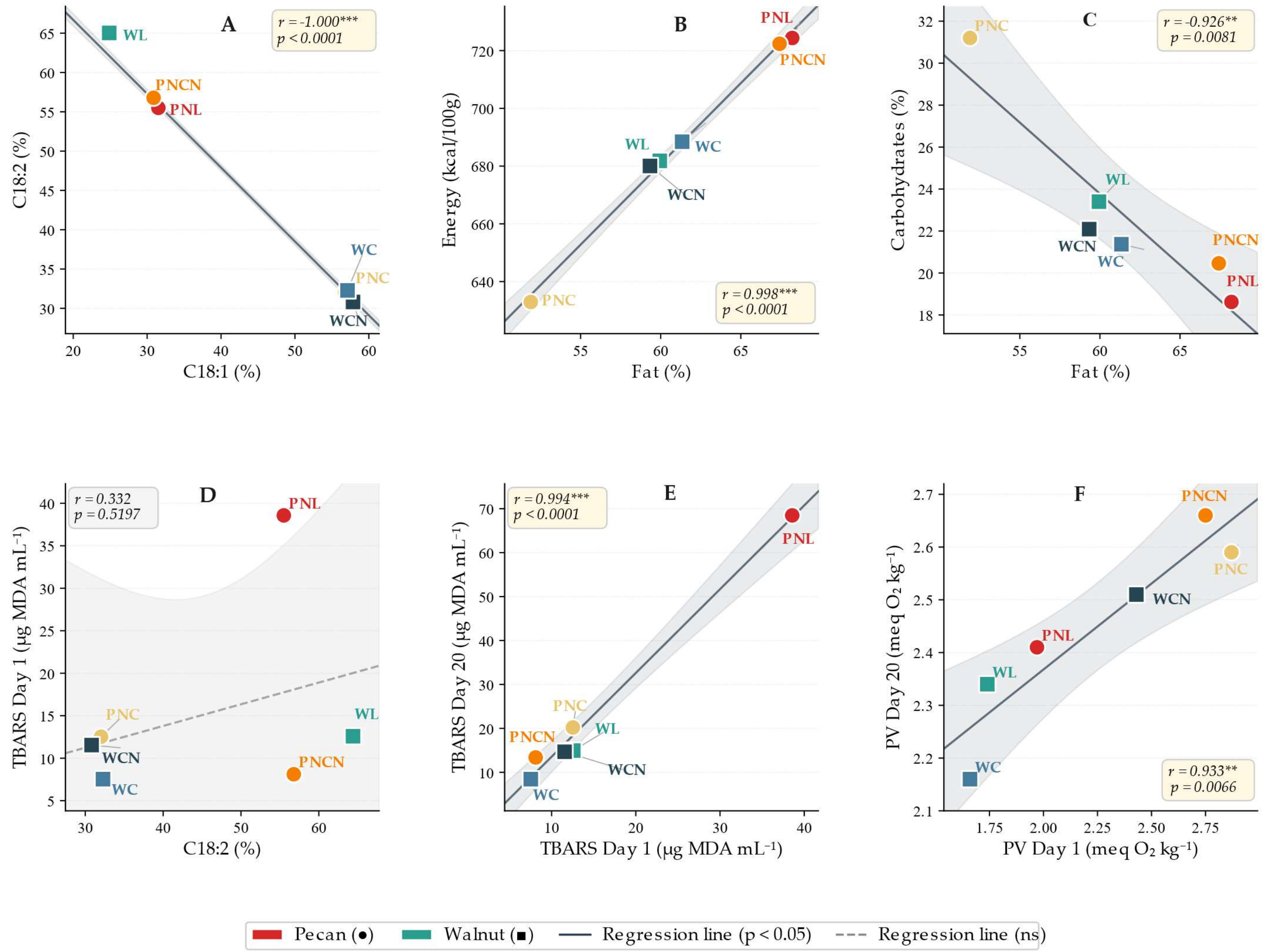

Figure S2. Bivariate scatter plots for six key variable pairs. Circles = pecan; squares = walnut. Solid lines: $p < 0.05$; dashed: not significant. Shaded areas = 95% CI. Yellow box = statistically significant. “PNL = pecan, Lovrin; PNCN = pecan, Cluj-Napoca; PNC = pecan, Chisinau; WL = walnut, Lovrin; WCN = walnut, Cluj-Napoca; WC = walnut, Chisinau.
